# Paradoxical role of β8 integrin on angiogenesis and vasculogenic mimicry in glioblastoma

**DOI:** 10.1038/s41419-022-04959-7

**Published:** 2022-06-08

**Authors:** Yang Liu, Xiangdong Xu, Yuxuan Zhang, Yunzhao Mo, Xinlin Sun, Lingling Shu, Yiquan Ke

**Affiliations:** 1 Department of Neuro-oncological Surgery, Zhujiang Hospital, Southern Medical University, Guangzhou, 510282 P. R. China; 2 The Neurosurgery Institute of Guangdong Province, Zhujiang Hospital, Southern Medical University, Guangzhou, 510282 P. R. China; 3 State Key Laboratory of Oncology in South China, Collaborative Innovation Center for Cancer Medicine, Sun Yat-sen University Cancer Center, Guangzhou, 510060 P. R. China; 4 Department of Hematological Oncology, Sun Yat-sen University Cancer Center, Guangzhou, 510060 P. R. China; 5 State Key Laboratory of Pharmaceutical Biotechnology, The University of Hong Kong, Hong Kong, P. R. China

**Keywords:** Cancer stem cells, CNS cancer

## Abstract

Glioblastoma multiforme (GBM) is the most aggressive and highly vascularized brain tumor with poor prognosis. Endothelial cell-dependent angiogenesis and tumor cell-dependent Vasculogenic mimicry (VM) synergistically contribute to glioma vascularization and progression. However, the mechanism underlying GBM vascularization remains unclear. In this study, GBM stem cells (GSCs) were divided into high and low β8 integrin (ITGB8) subpopulations. Co-culture assays followed by Cell Counting Kit-8 (CCK-8), migration, Matrigel tube formation, and sprouting assays were conducted to assess the proliferative, migratory and angiogenic capacity of GBM cells and human brain microvascular endothelial cells (hBMECs). An intracranial glioma model was constructed to assess the effect of ITGB8 on tumor vascularization in vivo. Our results indicated that ITGB8 expression was elevated in GSCs and positively associated with stem cell markers in glioma tissues, and could be induced by hypoxia and p38 activation. ITGB8 in GSCs inhibited the angiogenesis of hBMECs in vitro, while it promoted the ability of network formation and expression of VM-related proteins. The orthotopic GBM model showed that ITGB8 contributed to decreased angiogenesis, meanwhile enhanced invasiveness and VM formation. Mechanistic studies indicated that ITGB8-TGFβ1 axis modulates VM and epithelial-mesenchymal transition (EMT) process via Smad2/3-RhoA signaling. Together, our findings demonstrated a differential role for ITGB8 in the regulation of angiogenesis and VM formation in GBM, and suggest that pharmacological inhibition of ITGB8 may represent a promising therapeutic strategy for treatment of GBM.

## Introduction

Glioblastoma multiforme (GBM) is the most malignant brain tumor and is highly resistant to combination therapies [1]. Moreover, anti-angiogenic therapy has become a promising way to fight cancer [2]. However, in a phase II study of patients with newly diagnosed GBM, those administered with bevacizumab and temozolomide showed prolonged progression-free survival, while no improvement in overall survival [3, 4]. Therefore, further investigations of anti-angiogenic therapy in GBM are warranted.

Vasculogenic mimicry (VM) was firstly observed by Maniotis et al. in human melanoma cells and considered as a marker of aggressive tumor [5]. Analysis of xenograft models and human specimens unveiled that VM formation in patients with glioma usually predicts an unfavorable prognosis [6, 7]. Moreover, network formation in Matrigel has been widely used to evaluate the VM ability of tumor cells in vitro [8]. And various types of tumor cells are associated with tube formation in Matrigel under hypoxic condition [9–11]. Hence, VM has been considered as a compensation of angiogenesis, particularly in response to hypoxia. Moreover, VM acts as a novel paradigm for tumor perfusion, providing nutrition for tumor growth and progression.

Epithelial-mesenchymal transition (EMT), is also associated with tumor aggressiveness and metastasis [12]. Moreover, both VM and EMT could promote tumor cell motility and invasiveness. In addition, VM formation related signaling pathways including TGFβ, Notch, and Wnt, have also been shown to induce EMT process [13]. Genes involved in angiogenesis and vasculogenesis are upregulated in aggressive cancer cells, including cadherin-5 (CDH5), EPH receptor A2 (EPHA2) and laminin gamma2 (LAMC2) [14]. Matrix metallopeptidase 2 (mmp2) is also necessary in VM as it mediated extracellular matrix (ECM) remodeling via interacting with laminin 5γ2 chain [15]. During EMT process, epithelial markers (E-cadherin, cytokeratin) were downregulated while mesenchymal markers (N-cadherin, vimentin) were increased in tumor cells.

Recently cancer stem-like cells (CSCs) have been highlighted in malignant neoplasms for its role in chemotherapy resistance and recurrence [16, 17]. CSCs are a subpopulation within cancer cells that capable to self-renew and give rise to multiple cell lineages [18]. Recent studies demonstrated that a subpopulation of GBM cells could give rise to endothelial cells [19, 20]. Additionally, Hallani et al. and Scully et al. showed that some GSCs were able to transdifferentiate into smooth muscle cells or mural cells [21, 22]. Furthermore, Mani et al. illustrated that the induction of EMT in epithelial cells could lead to the elevation of stem cell markers [23].

Integrins belong to the family of a type I transmembrane heterodimeric glycoprotein receptors for ECM proteins [24]. β8 integrin (ITGB8) is crucial for the development of neuro-epithelial cells and microvasculature [25, 26]. ITGB8 was detected in multiple cancer types including lung adenocarcinoma, high grade serous ovarian cancer, gastric cancer and glioma, and correlated with poor survival [27–30]. Reyes et al. indicated that ITGB8 was overexpressed in GBM cells and correlated with diminished patient survival [27]. However, the effect of ITGB8 on VM and EMT remains unclear.

In the present study, we measured the dynamic changes of ITGB8 in GBM stem cells, and also investigated whether ITGB8 contribute to the VM formation in orthotopic GBM model. Furthermore, we explored the mechanisms whereby elevated ITGB8 triggers VM and EMT process through TGFβ1-Smad2/3-RhoA signaling pathway.

## Results

### β8 integrin is enriched in GSCs

**Fig. 1:**
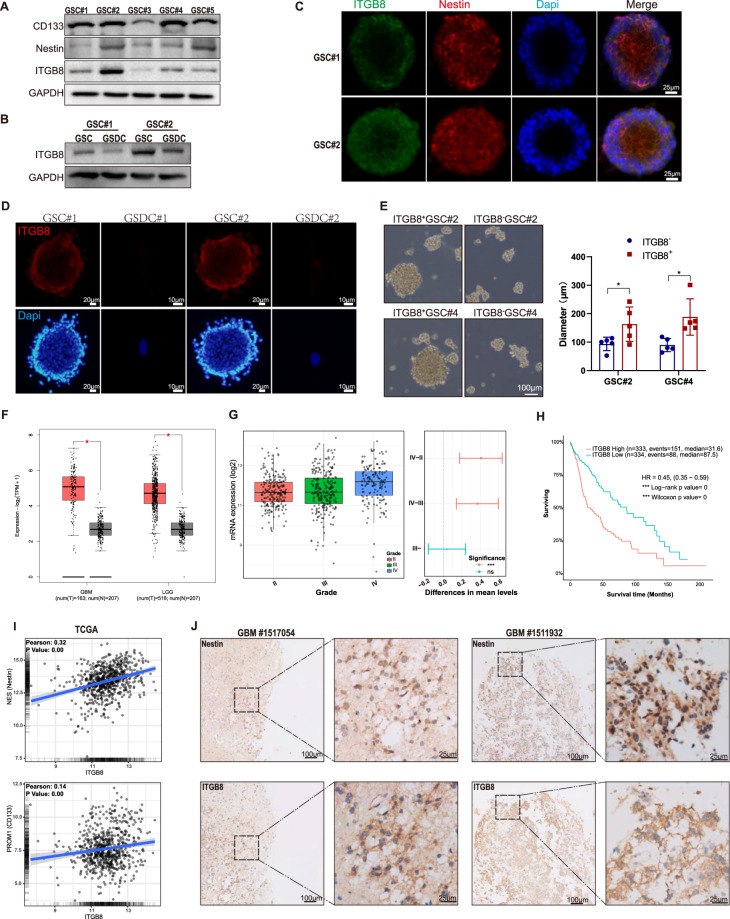
β8 integrin is enriched in GBM stem cells. **A** Levels of indicated proteins in five GBM stem cells derived from human GBM samples were determined via immunoblotting. **B** Protein levels of ITGB8 in GSCs and corresponding GSDCs were tested via immunoblotting. **C** Fluorescence staining for ITGB8 and Nestin in GSCs were evaluated. Scale bar = 25 μm. **D** Fluorescence staining for ITGB8 in GSCs and GSDCs were measured. Scale bar = 10/20 μm. **E** GSC#2 and GSC#4 fractionated into β8^−^ and β8^+^ cells, were cultured in serum-free medium to form floating spheres. Measurement of diameters was conducted in five randomly chosen neurospheres in each group. Scale bar = 100 μm. **F** Gene expression analysis of ITGB8 in GBM, LGG (TCGA) and normal brain tissues (GTEx) was conducted via GEPIA2 web tool. **G** Gene expression of ITGB8 in TCGA glioma dataset was analyzed based on WHO grade. **H** Kaplan–Meier analysis for overall survival of glioma patients with high or low ITGB8 expression in TCGA glioma datasets. **I** Correlation between gene expression of ITGB8 and stem cell markers in TCGA glioma dataset was evaluated. **J** Representative IHC images of Nestin and ITGB8 in human GBM tissues. Scale bar = 25/100 μm. Uncropped western blot images are shown in Supplementary Fig. 5. Data are expressed as mean ± SD.**p < 0.01, ***p < 0.001. IHC Immunohistochemistry.

We first examined ITGB8 expression in five human GSCs (GSC#1-GSC#5) and differentiated glioma cells. Immunoblot analysis revealed a robust expression of ITGB8, CD133 and Nestin in most of GSCs (Fig. 1A, C). GSCs were induced to differentiation in response to serum (10%) treatment. We next investigated the ITGB8 expression in GSCs serum-differentiated cells (GSDCs) and found that ITGB8 decreased significantly in GSDCs compared to that in GSCs (Fig. 1B, D). Additionally, β8^+^ GSCs showed robust self-renewal and proliferation capacities, while β8^-^ GSCs showed decreased capacity to form spheres (Fig. 1E). We further examined the mRNA expression of ITGB8 in mixed glioma dataset from The Cancer Genome Atlas (TCGA) database. ITGB8 expression was significantly elevated in GBM and LGG compared to normal brain tissue (Fig. 1F). Specifically, ITGB8 expression was upregulated in GBM (grade IV) tissue rather than that of grade II and III (Fig. 1G). Moreover, high levels of ITGB8 indicated poor prognosis in glioma dataset (Fig. 1H and S1b). CD133, Nestin and SOX2 expression were positively associated with ITGB8 expression in TCGA and Chinese Glioma Genome Atlas (CGGA) databases (Fig. 1I and S1c), and we further confirmed that ITGB8 was closely correlated with Nestin expression in GBM specimens (Fig. 1J). Together, our findings indicated that ITGB8 was abundant in GSCs.

### ITGB8 expression is regulated by hypoxia and p38 activation

**Fig. 2:**
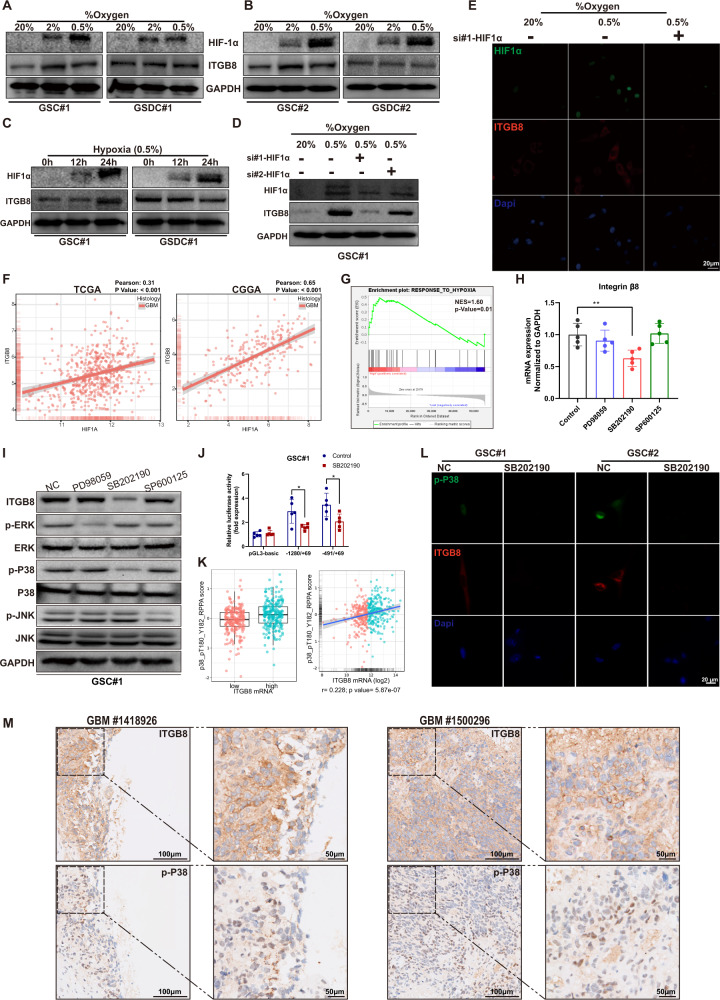
β8 integrin expression is mediated by hypoxia and p38 activation. **A**, **B** GSCs and corresponding GSDCs (GSCs serum-differentiated cells) were cultured in 20%, 2% or 0.5% O_2_ for 24 h. Protein levels of HIF1α and ITGB8 were analyzed using immunoblotting. **C** GSC#1 and GSDC#1 were exposed to 0.5% oxygen for 0, 12 or 24 h. Protein levels of HIF1α and ITGB8 were analyzed. **D** GSC#1 transfected with 2 siRNAs targeting HIF1α were cultured at 20% or 0.5% oxygen. And protein levels of HIF1α and ITGB8 were evaluated. **E** GSC#1 transfected with si-HIF1α or scrambled siRNA, were cultivated at 20% or 0.5% O_2_. Cells were then immunofluorescence stained for HIF1α and ITGB8. **F** Correlation between the expression of ITGB8 and HIF1α in TCGA or CGGA GBM dataset was assessed. **G** Correlation between ITGB8 expression and a hypoxia responsive gene set was determined via gene set enrichment analysis (GSEA). **H** GSC#2 was treated with MAPK inhibitors PD98059, SB202190 or SP600125, mRNA expression of ITGB8 was evaluated respectively. **I** Phosphorylated and total protein levels of ERK, p38 and JNK, as well as ITGB8 protein levels were measured in GSC#2 treated with various MAPK inhibitors. **J** Transcriptional activities of specific ITGB8 reporter constructs in the presence or absence of p38 inhibitor SB202190 were determined using luciferase reporter assay. **K** Association between p38 protein level and ITGB8 mRNA level was analyzed in TCGA glioma dataset. **L** Fluorescent staining for p-p38 and ITGB8 were performed in GSC#1 and GSC#2 with or without SB202190 treatment. **M** Representative IHC images of ITGB8, p-p38 in representative human GBM tissues. Uncropped western blot images are shown in Supplementary Fig. 5. Results are represented as mean ± SD of biologically triplicate assays. *p < 0.05, **p < 0.01, ***p < 0.001.

GSC#1 and GSC#2 were further selected for in vitro experiments due to the robust ITGB8 expression. To evaluate ITGB8 expression under hypoxic condition, GSCs and GSDCs were exposed to varying levels of O_2_ for 24 h. We found that hypoxia-induced Decreased O_2_ concentrations for 24 h led to the upregulation of β8 integrin in GSCs but not GSDCs (Fig. 2A, B). The protein level of ITGB8 increased accompanied with Hypoxia-inducible factor 1α (HIF1α) accumulation in GSCs, but remained unchanged in GSDCs (Fig. 2C). In line with this, expression of ITGB8 was significantly attenuated in GSCs treated with si-HIF1α (Fig. 2D). These results were further confirmed by immunofluorescence (Fig. 2E). Additionally, bioinformatics analysis revealed a positive association between ITGB8 and HIF1a expression in GBM datasets (Fig. 2F). Moreover, gene set enrichment analysis (GSEA) revealed that hypoxia-induced genes were significantly correlated to ITGB8 in GBM (Fig. 2G).

As integrins were widely regulated by MAPK pathways in both tumors and normal tissues [31, 32]. GSCs#2 was treated with inhibitors targeting ERK (PD98059), P38 (SB202190) and JNK (SP600125) to examine ITGB8 expression in relation to these pathways. mRNA and protein levels of ITGB8 were mostly affected by p38 signaling (Fig. 2H, I and S2c). Previous study has shown that ITGB8 promoter region was located in area from −1280 to 69 bp of gene sequence with multiple putative transcription factor binding site (Fig. S2a) [24]. We performed a luciferase reporter assay with various ITGB8 reporter constructs. The region from −491 to 69 bp was critical for transcriptional regulation of ITGB8 in GBM stem cells (Fig. 2J and S2d). Furthermore, a significant correlation between ITGB8 mRNA abundance and p38 phosphorylation was observed in TCGA glioma dataset (Fig. 2K). Furthermore, ITGB8 expression significantly decreased in GSCs pretreated with P38 inhibitor (Fig. 2L), and this was further confirmed by immunohistochemistry (IHC) staining in GBM samples (Fig. 2M). In summary, ITGB8 expression was regulated in GBM in response to hypoxia and p38 activation.

### ITGB8 correlates with reduced angiogenesis

**Fig. 3:**
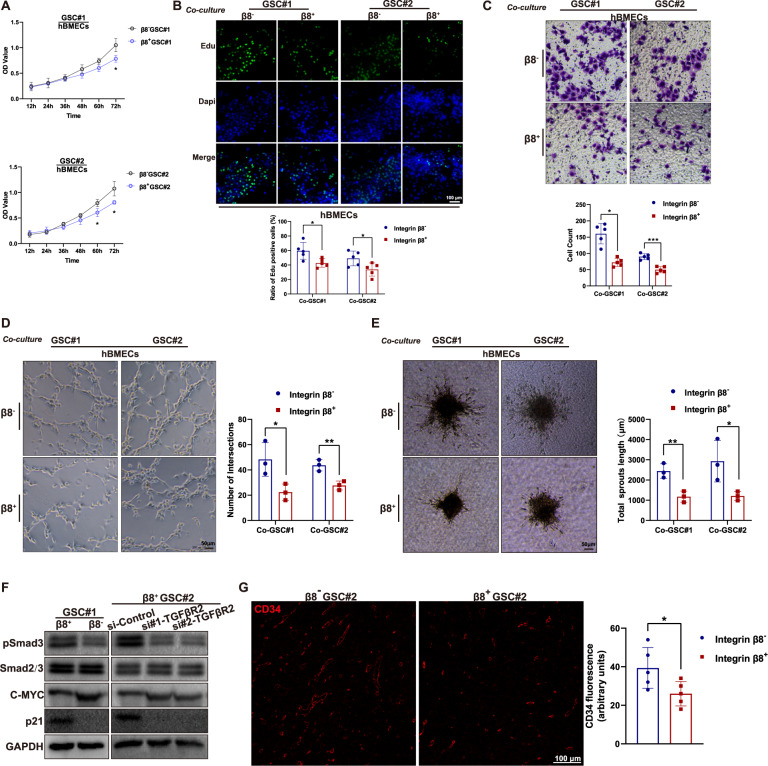
GSCs-derived β8 integrin expression inhibits angiogenic capacity of human brain microvascular endothelium. **A** hBMECs were co-cultured with β8^−^ or β8^+^ GSCs for 24 h, cell proliferation was determined via CCK-8 assay. **B** hBMECs co-cultured with β8^−^ or β8^+^ GSCs were subjected to Edu staining. Scale bar = 100 μm. **C** Migration assay was conducted of hBMECs that co-cultured with β8^−^ or β8^+^ GSCs. **D** Tube formation assay on Matrigel was performed of hBMECs that co-cultured with β8^−^ or β8^+^ GSCs. Scale bar = 50 μm. **E** hBMECs were co-cultured with β8^−^ or β8^+^ GSCs. Spheroid-based angiogenesis assay was performed. **F** The protein levels of phosphorylated Smads, c-Myc and p21 in hBMECs cultured with β8^−^ or β8^+^ GSC#1 (left panel). Indicated proteins in hBMECs treated with scrambled siRNA or siTGFβR2, followed by co-culture with GSC#2. **G** Orthotopic xenografts GBM model was established with β8^−^ or β8^+^ GSC#2. Sections of five random mice of each group were selected and immunofluorescence stained for CD34. Scale bar = 100 μm. Uncropped western blot images are shown in Supplementary Fig. 5. Results are represented as mean ± SD of biologically triplicate assays. *p < 0.05, **p < 0.01, ***p < 0.001.

To explore the potential interaction between GSCs and human brain microvascular endothelial cells (hBMECs), GSCs were co-cultured with hBMECs under different conditions (Fig. S3a). hBMECs co-cultured with β8^+^GSCs showed diminished proliferative capacity compared to those with β8^−^ GSCs (Fig. 3A, B). Migration assay showed that the number of hBMECs crossed the Matrigel layer toward β8^+^ GSCs was significantly lower than that toward β8^−^ GSCs (Fig. 3C). Next, hBMECs were seeded on Matrigel-coated lower plates to investigate the tube formation (Fig. S3b). The hBMECs co-cultured with β8^+^ GSCs showed attenuated network formation compared to those with β8^−^ GSCs (Fig. 3D). Moreover, we established an in vitro angiogenesis model, in which hBMEC spheroids were located in the lower chamber with collagen I solution (Fig. S3c). An inhibited capacity of hBMECs spheroid-based angiogenesis was observed when co-cultured with β8^+^GSCs compared to that with β8^−^GSCs (Fig. 3E). The proliferation of hBMECs significantly decreased when cultured in conditioned medium from β8^+^GSCs (Fig. S4a-c), furthermore, tube formation (Fig. S4d) and sprouting angiogenesis (Fig. S4e) were also attenuated when compared to that of hBMECs cultured with β8^−^GSCs.

As ITGB8-TGFβ signaling contributes greatly to endothelial cells development of retinal [33], we co-cultured hBMECs with β8^+^ or β8^−^ GSC#1. The protein levels of phosphorylated Smad, and p21 were elevated in brain endothelial cells cultured with β8^+^ GSC#1 while c-myc expression was decreased accordingly (Fig. 3F). We also established orthotopic xenografts in nude mice with β8^+^ or β8^−^ GSC#2, and the vessel quantification revealed an increased angiogenesis in β8^−^ GSC#2 intracranial tumors comparing to that of β8^+^ GSC#2 (Fig. 3G). Together, these findings suggested that ITGB8 alleviated angiogenesis in GBM.

### ITGB8 induces VM and EMT process

**Fig. 4:**
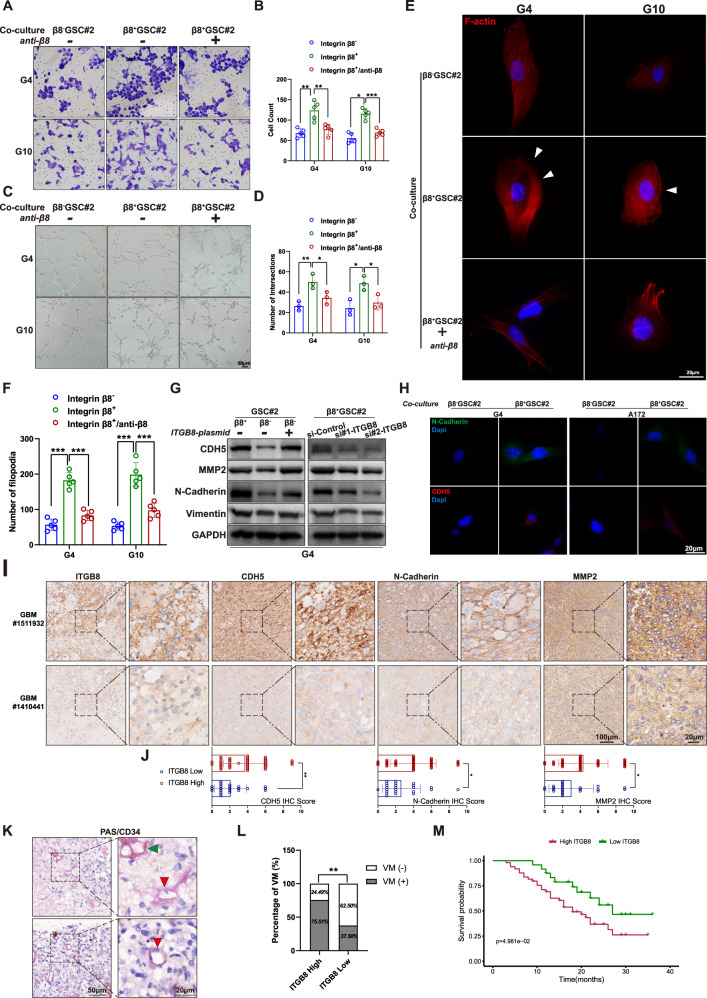
β8 integrin expressed in GSCs induces elevated network formation and invasive phenotype of GBM cells. **A** Primary GBM cells were untreated or pretreated with blocking antibody targeting β8 integrin and co-cultured with β8^−^ or β8^+^ GSC#2. Migration abilities of GBM cells were determined. **B** Migrated cells were quantified. **C**, **D** Primary GBM cells treated in **A** were cultured on Matrigel. Network formation capacities of GBM cells were calculated. **E** Primary GBM cells treated and co-cultured in **A**, were subjected to immunofluorescence staining with phalloidin. Scale bar = 20 μm. **F** Filopodia number in **E** was quantitated and analyzed. **G** In the left panel, β8^+^ and β8^−^ GSC#2 were transfected with ITGB8-plasmid or empty vector. In the right panel, β8^+^ GSC#2 was transfected with scrambled negative control siRNA or siRNA targeting ITGB8. Protein levels of CDH5, MMP2, N-Cadherin and Vimentin were measured. **H** GBM cells G4 and A172 were co-cultured with β8^+^ or β8^−^ GSC#2. N-Cadherin and CDH5 expression were determined by immunofluorescence staining. Scale bar = 20 μm. **I** IHC staining of ITGB8, CDH5, N-Cadherin and MMP2 in representative human GBM sections. Scale bar = 20/100 μm. **J** Bar charts presenting IHC score of CDH5, N-Cadherin and MMP2 in ITGB8 low or high GBM samples, revealed associations between the expression of ITGB8 and indicated proteins. **K** VM pattern was identified by PAS/CD34 dual staining. Green arrowhead indicates CD34^+^ endothelial-based vascular channel, while red arrowhead indicates CD34^-^ VM channel. IHC image in the lower right panel presents a vascular lumen, around which CD34 staining is absent and a deformed nucleus is indicated. **L** VM pattern quantitation and analysis in human GBM samples with high or low ITGB8 expression. **M** Kaplan–Meier survival plot of GBM patients with high or low ITGB8 expression. Uncropped western blot images are shown in Supplementary Fig. 5. Results are represented as mean ± SD of biologically triplicate assays. *p < 0.05, **p < 0.01, ***p < 0.001. ns not significant.

Primary GBM cells and A172 cells showed enhanced migration when co-cultivated with β8^+^ GSCs rather than β8^−^ GSCs and neutralizing antibody treated-GSCs (Fig. S3d, Fig. 4A, B). GSC#2 was added into the upper insert and G4 was seeded on Matrigel in the lower plate, and the tube formation ability of GBM cells was evaluated (Fig. S3e). Tubule formation of G4 increased significantly when co-cultured with β8^+^ GSC#2, which was alleviated by β8-neutralizing antibody pretreatment (Fig. 4C, D). Tumor cells undergo EMT would display a remodeling of actin [34]. G4 and G10 cells co-cultured with β8^+^ GSC#2 showed increased filopodia formation, which was attenuated when GSCs were pretreated with β8-neutralizing antibody (Fig. 4E, F). Immunoblot analysis revealed augmented CDH5, MMP2, N-Cadherin and vimentin expression in GBM cells when co-cultivated with β8^+^ GSCs. And forced expression of ITGB8 in β8^−^ GSCs led to the upregulation of these molecules in co-cultured GBM cells. Meanwhile, downregulating ITGB8 in β8^+^ GSC#2 caused declined expression of CDH5, MMP2, N-Cadherin and vimentin (Fig. 4G). These results were further confirmed by ICC and IHC staining analysis (Fig. 4H–J). Additionally, we performed PAS/CD34 dual staining to detect VM in GBM tissues (Fig. 4K). A classic VM pattern was characterized as PAS-positive and CD34-negative [35]. VM quantity was significantly associated with ITGB8 expression (Fig. 4L). In addition, survival analysis demonstrated a positive association between ITGB8 expression and adverse outcomes (Fig. 4M).

### ITGB8 modulates VM formation and invasion of glioblastoma cells through TGFβ-RhoA signaling

**Fig. 5:**
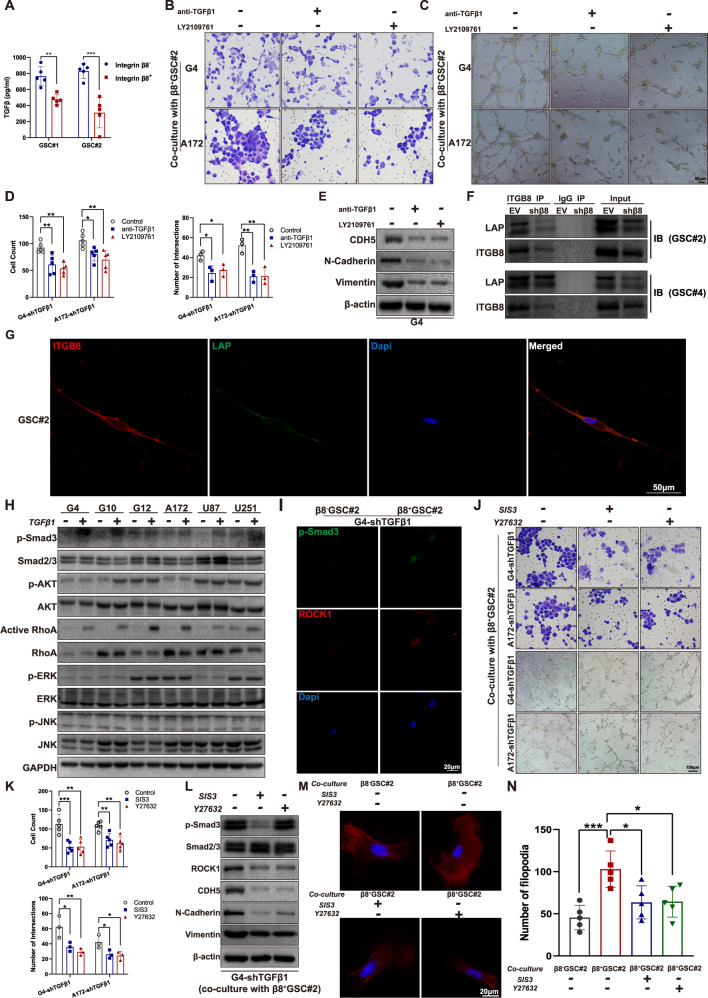
β8 integrin regulates VM formation and invasive phenotype of GBM cells via activating TGFβ1/p-smad3/RhoA signaling. **A** Levels of secreted TGFβ1 in culture medium of GSC#1 and GSC#2. **B** Migration abilities of GBM cells co-cultured with β8^+^ GSCs were assessed with the addition of neutralizing antibody targeting TGFβ1 or TGFβ receptor inhibitor LY2109761. **C** Tube formation capacities of GBM cells treated in **B** were assessed. **D** Statistical analysis of migration and tube formation assay. **E** Protein levels of CDH5, N-Cadherin and Vimentin in G4 cells were measured via immunoblotting. **F** Co-IP of ITGB8 and LAP (TGFβ1) in GSC#s analyzed by immunoblotting. **G** Confocal images of immunofluorescence staining for ITGB8 (red), LAP (green) and dapi (blue) in GSC#2 revealed co-localization of ITGB8 and LAP. Scale bar = 50 μm. **H** Six GBM cells were untreated or treated with 100 pM TGFβ1 for 6 h, indicated protein levels were determined by immunoblotting. **I** Immunofluorescence staining of p-Smad3 and ROCK1 in G4-shTGFβ1 cells co-cultured with β8^+^ or β8^−^ GSC#2. **J** GBM cells G4-shTGFβ1 and A172-shTGFβ1 were treated with SIS3 (3 μM) and Y27632 (10 μM) for 1 h, followed by co-culturing with β8^+^ GSC#2 for 12 h. Migration and tube formation capacities were then determined. **K** Statistical analysis of migration and tube formation assay. **L** G4-shTGFβ1 was treated under same condition described above. Levels of p-Smad3, Smad2/3, ROCK1, CDH5, N-Cadherin and Vimentin were measured via immunoblotting. **M** G4-shTGFβ1 cells were pretreated with SIS3 (3 μM) or Y27632 (10 μM) for 1 h and subsequently co-cultured with β8^+^ or β8^−^ GSC#2 respectively. Immunofluorescence staining for F-actin was conducted with phalloidin. Scale bar = 50 μm. **N** Filopodia quantitation in G4-shTGFβ1 was measured and statistically analyzed. Uncropped western blot images are shown in Supplementary Fig. 6. Results are represented as mean ± SD of biologically triplicate assays. *p < 0.05, **p < 0.01, ***p < 0.001. ns not significant.

Previous studies revealed ITGB8/ TGFβ axis as a prominent angiogenesis regulator during CNS development [36, 37]. β8^+^ GSCs produced more abundant TGFβ1 in culture medium comparing to that of β8^−^ GSCs (Fig. 5A). We transfected glioma cells with shTGFβ to eliminate the autocrine effect of TGFβ. G4-shTGFβand A172-shTGFβ, pretreated with neutralizing antibody targeting TGFβ1 or LY2109761 (an inhibitor of the TβRI), were co-cultured with β8^+^ GSCs and subsequently subjected to migration and tube formation assay analysis. TGFβ1 blockage or TGFβ receptor inactivation led to attenuated migratory and tube formation capacities (Fig. 5B–D). It also significantly decreased the expression of CDH5, N-Cadherin and vimentin (Fig. 5E). TGFβ is released in a latent complex containing TGFβ, latency associated protein (LAP), and a latent TGFβ binding protein (LTBP) [38]. Previous experiments have shown that ITGB8 is expressed on the surface of astrocyte and bind to the LAP of TGFβ1 in perivascular astrocyte and T lymphocyte [39, 40]. We conducted an immunoprecipitation assay in GSCs transfected with control vector or sh-β8 integrin. GSC#2 and GSC#4 expressing ITGB8 were detected with co-immunoprecipitated latent TGFβ1 (Fig. 5F). Confocal imaging analysis revealed a co-localization of ITGB8 and latent TGFβ1 on the cell membrane of GSC#2 (Fig. 5G). The cellular level of p-Smad3 as an indicator of the canonical TGFβ-smad2/3 pathways, and p-Akt, active RhoA, p-ERK, p-JNK as indicators of non-canonical TGFβ pathways in GBM cells treated with exogenous TGFβ were evaluated. Interestingly, elevated expression of p-Smad3, and activated RhoA was observed in response to TGFβ stimulation (Fig. 5H). P-Smad3 and Rho-associated coiled-coil-containing protein kinase 1 (ROCK1) expression in G4-shTGFβ1 cells co-cultured with β8^−^ or β8^+^ GSC#2 positively correlated with β8 integrin expression (Fig. 5I). To further illustrate the role of Smad2/3 and RhoA signaling in regulating ITGB8-induced invasive phenotype and VM formation, we pretreated TGFβ1 knock-out G4 and A172 cells with SIS3 (a specific inhibitor of Smad3 phosphorylation) or Y27632 (a RhoA kinase inhibitor), followed by co-cultivation with β8^+^ GSCs. The migration and tube formation capacities in G4-shTGFβ1 cells were significantly decreased (Fig. 5J, K). Inhibition of Smad3 phosphorylation or RhoA kinase also decreased the expression of CDH5, N-Cadherin and vimentin (Fig. 5L). F-actin staining revealed a similar effect of SIS3 and Y27632 on cytoskeleton alteration. Filopodia formation in G4-shTGFβ1 cells increased when co-cultured with β8^+^ GSC#2 than that with β8^−^ GSC#2, however, it was attenuated when treated with SIS3 or Y27632 (Fig. 5M, N). Taken together, these findings suggested that GSCs-derived ITGB8 regulated GBM cells by mediating TGFβ/Smad/RhoA signaling pathway.

### ITGB8 is associated with VM in an intracranial xenograft model

**Fig. 6:**
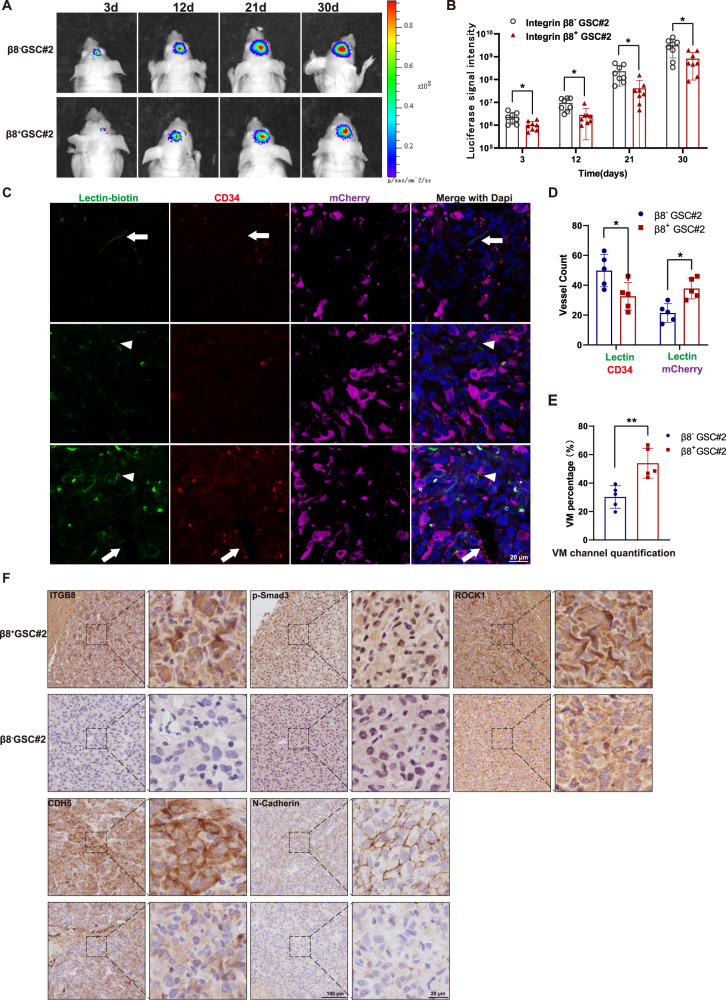
β8 integrin contributes to VM formation in intracranial GBM xenograft. **A** Luminescent imaging of representative nude mice xenografts from mCherry-labeled β8^+^ (*n* = 8) or β8^−^ GSC#2 (*n* = 8) at day 3, 12, 21 and 30. **B** Luminescent signal intensity of GBM-bearing mice in two groups were evaluated. **C** Representative immunofluorescence images of vascular channels lined by ECs or tumor cells. Arrows indicate the regular CD34^+^ vessels, while arrowheads indicate CD34^-^/Lectin^+^ vessels. Scale bar = 20 μm. **D** Quantification of lectin (+) vessels stained positively by CD34 or mCherry. **E** Percentage of VM vessels in β8^+^ or β8^−^ integrin xenografts. **F** IHC staining of ITGB8, p-Smad3, ROCK1, CDH5 and N-Cadherin in β8^+^ and β8^−^ integrin xenografts. Results are represented as mean ± SD of biologically triplicate assays. *p < 0.05, **p < 0.01, ***p < 0.001.

To explore ITGB8-mediated angiogenesis and VM in vivo, we established a xenograft model by orthotopically implanting mCherry-labeled β8^−^ or β8^+^ GSC#2 into nude mice. Mice with β8^+^ GSCs exhibited a decreased tumor growth rate when compared to mice implanted with β8^−^ GSC#2 (Fig. 6A, B). The endothelium-based vascular channel presented positive lectin and CD34 staining, while VM channel was characterized by positive lectin and mCherry staining (Fig. 6C). These results indicated that the VM formation in β8^+^ GSCs-xenograft GBM was significantly enhanced (Fig. 6D, E). Furthermore, the β8^+^ GSCs-xenograft was positively correlated with augmented expression of β8 integrin, p-Smad3 and ROCK1, as well as indicators for VM and EMT (Fig. 6F). In summary, these findings indicated that ITGB8 plays a critical role in VM formation of GBM in vivo.

## Discussion

**Fig. 7:**
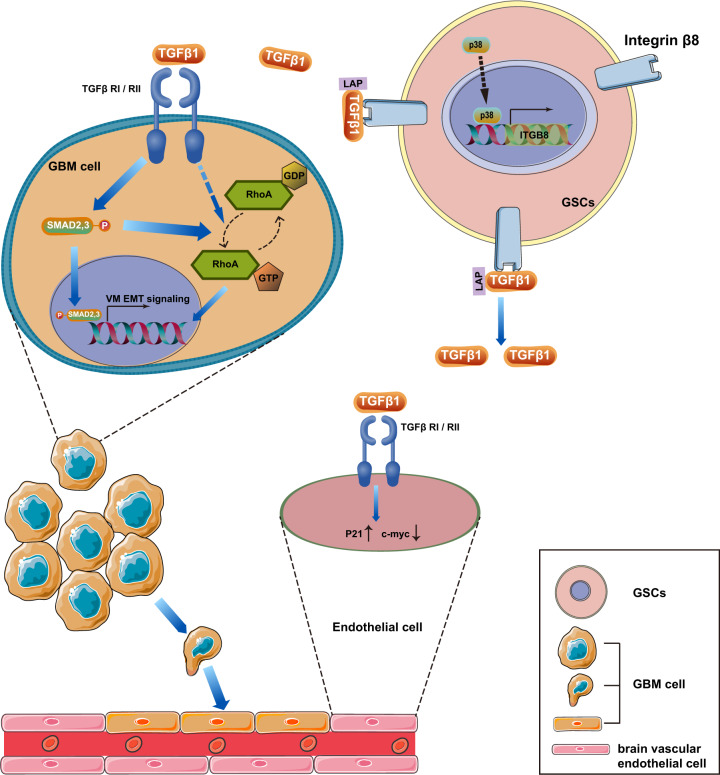
Schematic diagram illustrating the β8 integrin-TGFβ1 axis in VM regulation. GSCs-derived β8 integrin, which was regulated by p38 activation, released secreted TGFβ1 into GBM microenvironment. GBM cells exhibit activated p-Smad and RhoA and eventually induce VM and EMT phenotype. While human brain microvascular endothelial cells presented upregulated p21 and decreased c-myc expression that ultimately culminate in the suppression of GBM angiogenesis.

In the present study, we provided clinical, in vitro and animal evidences demonstrating that GSCs-derived ITGB8 exhibits anti-angiogenic effect on brain microvascular endothelial cells, and contributes to VM formation and EMT in GBM to support tumor invasion. Mechanistic investigation indicated that ITGB8 promotes VM formation and invasive phenotype in GBM cells via activating the TGFβ1/p-Smad/RhoA signaling pathway (Fig. 7).

Our results revealed that cancer stem-like cells derived from primary GBM displayed abundant ITGB8 expression. Moreover, we detected the loss of ITGB8 in GSCs with serum-induced differentiation in vitro. In fact, ITGB8 has been reported to be expressed in neural stem and progenitor cells of adult mouse brain [26, 41]. Similar to stem or progenitor cells, cancer stem cells possess the ability of self-renewal and differentiation and are crucial for tumor progression and metastasis [42]. ITGB8 was recently reported to be upregulated in various types of aggressive tumors. Mertens-Walker et al. reported that ITGB8 expression was positively associated with EphB4 receptor tyrosine kinase in prostate cancer cells and played an important role in tumor cell motility [43]. Jin et al. revealed ITGB8 as a determinant of pancreatic ductal adenocarcinoma radiochemosensitivity [44]. Additionally, overexpression of ITGB8 led to the growth and metastasis of colorectal cancer [45]. Moreover, expression of ITGB8 contributed to unfavorable prognosis of high grade serous ovarian cancers [30]. Our data showed that ITGB8 in GSCs, but not differentiated glioma cells, could be induced by hypoxia (Fig. 2A, B). Low oxygen tension facilitated the maintenance of undifferentiated states of embryonic and neural stem cell phenotypes [46]. In fact, previous studies have shown that ITGB8 is partly co-expressed with other stem cell markers such as CD133 [47].

ITGB8 in neuro-epithelial cells and astrocytes is crucial for cerebral angiogenesis and development [26, 36]. For instance, Mobley and colleagues showed that β8^-/-^ mice displayed compromised blood-brain barrier properties [26]. However, the effects of ITGB8 on tumor neovascularization are much more complex. Takasaka et al. reported that β8 integrin expression was associated with increased angiogenesis and a specific ITGB8 blocking antibody substantially diminished vessel density in mouse xenografts derived from MC38 colon carcinoma cancer cell line [48]. While Fang et al. suggested that reduced ITGB8 expression in glioma cells favored angiogenesis [49]. Our data implied that enriched ITGB8 in GSCs accounts for reduced GBM angiogenesis. VM contributes to the failure of anti-VEGF therapy in solid cancers, including GBM [50]. We presented evidence in current study supporting the importance of β8 integrin in GSCs-mediated VM formation.

Mechanical investigations demonstrated that activation of the TGFβ/Smad signaling pathway is involved in ITGB8-mediated VM formation. Latent TGFβ1 is expressed in diverse tumor cells and can be activated by binding to ITGB8 [51]. Our work suggested that the expression of ITGB8 in GSCs was positively correlated with TGFβ1 levels. In addition to the Smad-dependent pathway, non-Smad pathways including MAPK, Rho-like GTPase and PI3K/AKT signaling pathways have been reported in carcinogenesis and tumor progression [51–53]. Our study showed that ITGB8-TGFβ1 affected downstream activation of Smad2/3 and RhoA in glioma cells. Attenuation of VM or invasive phenotype may result from inhibition of the Smad or RhoA pathway.

We further established orthotopic GBM models in nude mice with either integrin β8^+^ or β8^−^ primary GSCs. Mice in β8^+^ group were associated with increased VM formation and EMT. Since ITGB8 caused opposite prognosis in patients with GBM, we speculated that endothelial-based angiogenesis mainly accounts for the outcome and tumor progression in GBM-bearing mice when no therapeutic intervention was received. While VM may play a greater role in GBM patients that clinically undergo surgery or chemotherapy.

## Conclusion

In summary, our study depicted a complex role of β8 integrin in glioma vascularization. We provided evidence that β8 integrin expression was inversely correlated with angiogenesis, while promoted VM formation via inducing TGFβ expression, and subsequently activating the RhoA signaling pathway. Therefore, β8 integrin may be a potential therapeutic target for GBM VM and invasion. Pharmacological inhibition of β8 integrin together with anti-VEGF agent would effectively suppress tumor vascularization and prolong survival of patients with GBM.

## Materials and methods

### GBM specimen and cell culture

Brain tumor samples were obtained from consenting patients diagnosed as GBM. 73 paraffin-embedded GBM samples and corresponding clinicopathological data were collected from patients undergoing surgical operation in the department of neurosurgery at Zhujiang Hospital from 2013 to 2017.

Glioblastoma tissues were enzymatically digested and GBM stem cells were cultured in DMEM/F12 (Gibco, USA) medium supplemented with EGF (20 ng/ml, Peprotech, USA), bFGF (20 ng/ml, Peprotech) and B27 (1:50, Gibco).

### Reagents, plasmids construction and siRNA

ITGB8 cDNA and plasmids construction, as well as siRNAs for target genes were designed and provided by Sangon Biological Engineering Technology and Service Co., Ltd. (Shanghai, P.R. China). PCR primer sequences for IGFB8 and GAPDH were provided in supplementary table 2.

### Sphere formation assay

Dissociated single GBM stem cells were seeded into 24-well plates and incubated in serum-free medium at 37 °C for 7 days. Diameters of 5 randomly selected tumor spheres were measured.

### Cell proliferation and migration assay

Cell proliferation was measured via using CCK-8 and Edu assay. Migration assay was performed by using cell culture insert with 8-um pores in 24-well plates (Costar, USA).

### Tube formation assay

Tube formation assay of glioma cells and hBMECs was carried out as previously described [6]. Cells were cultured on Matrigel (BD Biosciences, USA) for 24 h and tubules were quantified.

### Endothelium spheroid-based sprouting angiogenesis assay

In vitro angiogenesis assay was performed according to methods previously published by Korff and colleagues with minor modification [54]. Detailed procedures were described in Supplementary methods.

### Immunological analysis

Human TGF-beta1 ELISA kit (Proteintech) was used to measure the concentration of TGF-beta1 from GSCs-derived culture media according to manufacturer’s instruction.

### Immunoblot Analysis and Immunoprecipitation assays

The immunoblot and immunoprecipitation assay were performed as described before [6]. Lysates from certain cells were subjected to immunoblot analysis using antibodies which is listed in supplementary table 2.

### Tissue immunohistochemical and immunofluorescence staining

Tissue IHC and IF staining were performed as previously reported. Briefly, specimens of surgical GBM tissues and xenograft samples were fixed, embedded and sectioned followed by immuno-staining. Antibodies used for targeted proteins were listed in supplementary table 2.

### Luciferase reporter assay

Luciferase reporter assay was carried out according to the protocol described previously [55]. Luciferase activity was measured using dual-luciferase reporter assay kit with Renilla luciferase activity as control (Promega, Mannheim, Germany).

### In vivo xenograft assay

Five to eight-week-old Balb/c male mice were purchased from the Central Animal Facility of Southern Medical University. Fractionated β8^+^ or β8^−^ GSCs cells (1 × 105 cells in 0.1 ml PBS) stably transfected with mCherry-LUC vector were orthotopically injected into the brain of Balb/c nude mice according to Ozawa’s instruction [56]. Each group included 8 mice.

### Vasculature quantification

Vasculature quantification was measured according to the method previously reported [57]. Three random specimens from each xenograft sample were subjected to CD34, mCherry and lectin (i.v.) staining. Lectin^+^/mCherry^+^ lumens stand for VM vessels and CD34^+^ lumens stand for regular endothelium-based vessels. ImageJ software was used for vessel density quantification.

### Statistical analysis

All statistical analyses in this study were performed using Prism 8.0 (GraphPad Software Inc., USA) and R software. Data were expressed as mean ± SD. Sample size for each study was determined based on literature documentation of similar well-characterized experiments. Statistical significance was assessed by Student’s *t*-test or one-way ANOVA with Bonferroni correction for multiple comparisons. P value smaller than 0.05 was considered statistically significant. Statistical outlier analysis was calculated using the GraphPad Outlier calculator. Those significant outliers were excluded from data analysis.

## Supplementary information

Supplementary figures and tables

Reproducibility checklist

## Data Availability

All data generated during this study are included either in the main article or in the supplementary information files.
