# Supplementary material for: Paradoxical role of β8 integrin on angiogenesis and vasculogenic mimicry in glioblastoma
Source: Cell Death Dis. 2022 Jun 8;13(6):536. doi: 10.1038/s41419-022-04959-7 (PMC9177864; doi:10.1038/s41419-022-04959-7)
Supplement: Supplementary file 1 — Supplementary figures and tables [file 41419_2022_4959_MOESM1_ESM.pdf]

## Supplementary Figures

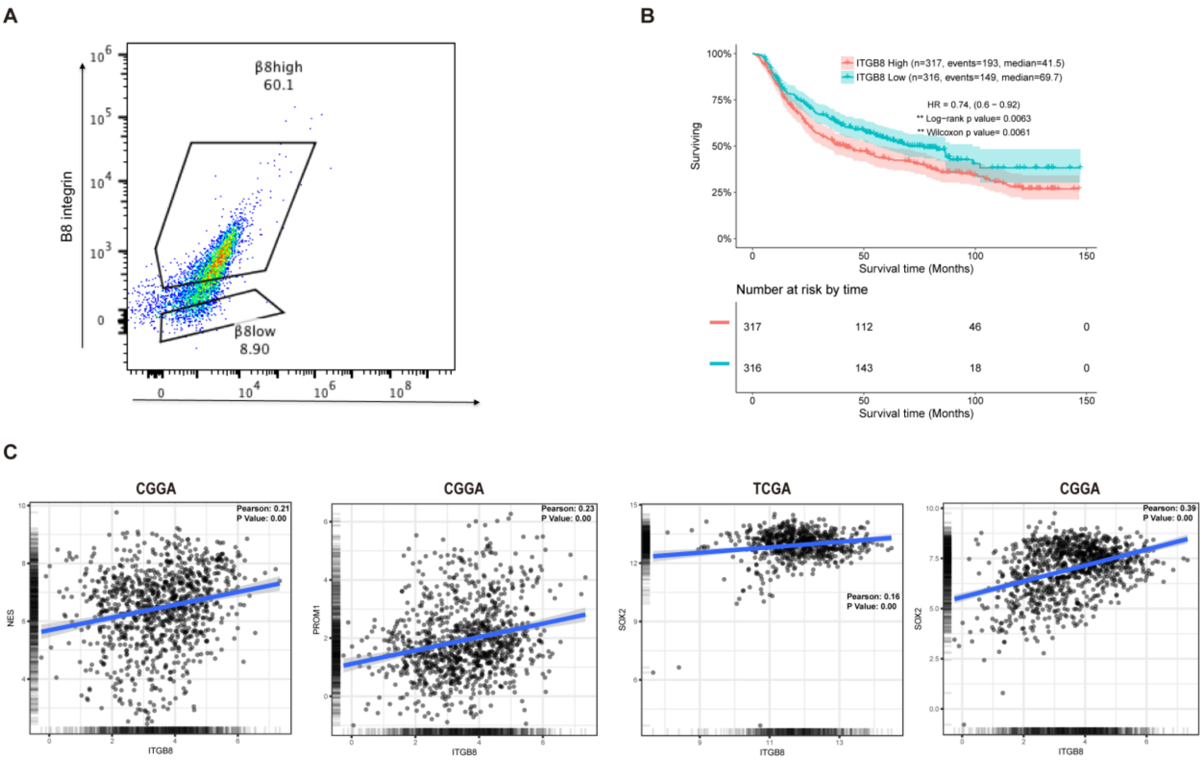


**Figure S1. Expression of β8 integrin in GSCs.** (**a**) Primary GSCs were fractionated into high β8 integrin ($\beta8^+$) and low β8 integrin ($\beta8^-$) cells through FACS. (**b**) Kaplan–Meier analysis for overall survival of glioma patients in CGGA based on β8 integrin expression. (**c**) correlation between expression of ITGB8 and neural stem cell markers including Nestin, CD133 and SOX2 in TCGA and CGGA glioma datasets. *p< 0.05, **p< 0.01, ***p< 0.001. ns, not significant.

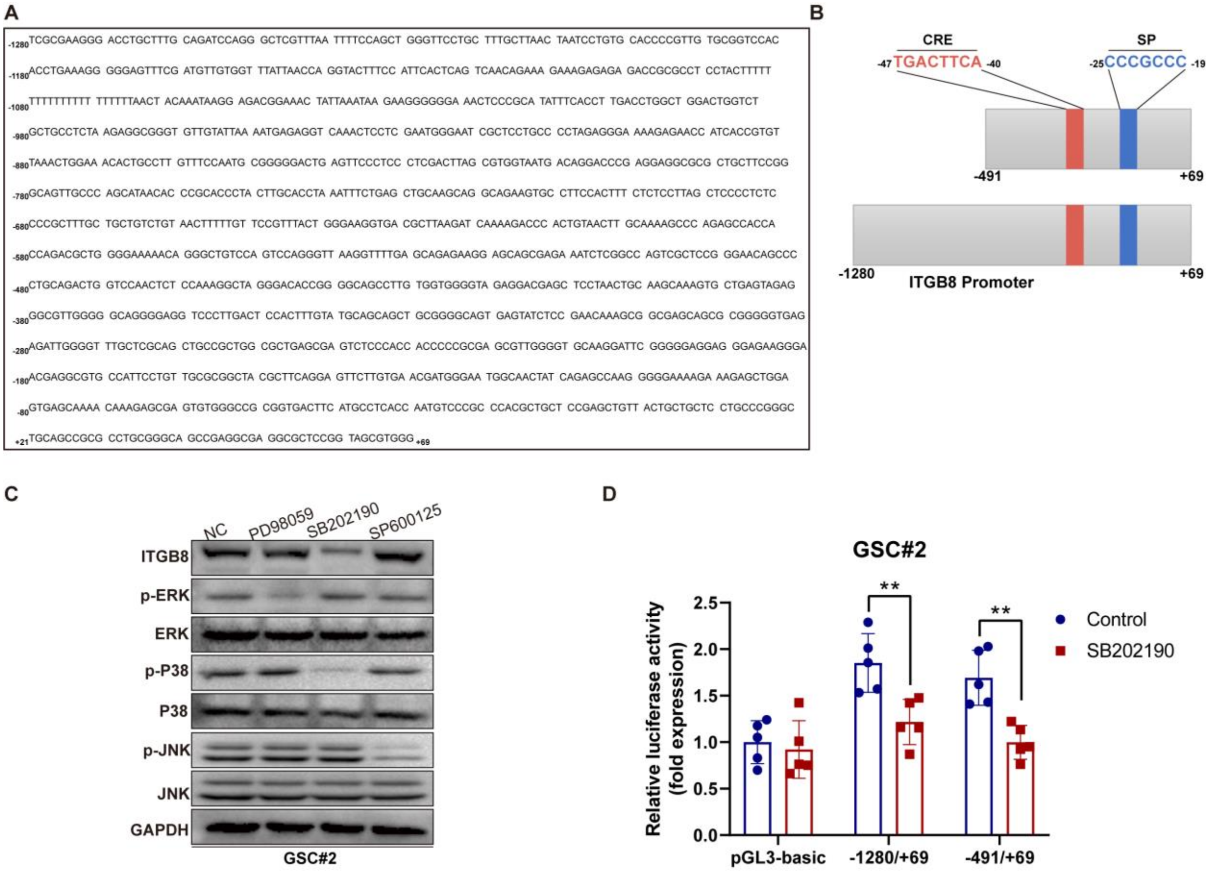


**Figure S2. ITGB8 promoter was regulated by p38 activation.** (**a**) Sequence of ITGB8 promoter region from -1280 to 190 bp. (**b**) Specific ITGB8 reporter constructs and putative transcription factor binding sites were presented. (**c**) Phosphorylated and total protein levels of ERK, p38 and JNK was measured in GSC#2 treated with MAPK inhibitors. (**d**) Transcriptional activities of specific ITGB8 reporter constructs in the presence or absence of p38 inhibitor SB202190 were determined using luciferase reporter assay. Uncropped western blot images are shown in Supplementary Fig. 6. Results are represented as Mean ± SD of biologically triplicate assays. *p< 0.05, **p< 0.01, ***p< 0.001.

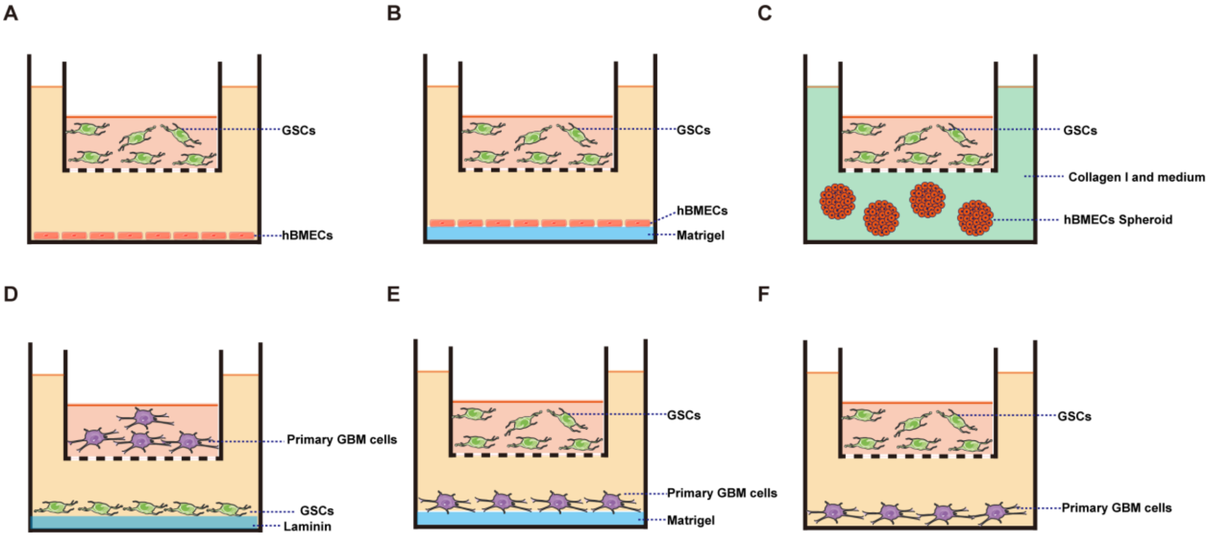


**Figure S3. Co-culture model used in this study.** (**a**) GSCs were seeded onto the upper insert, while hBMECs were cultured in the lower plates. (**b**) GSCs were seeded onto the upper insert, and hBMECs were cultured on Matrigel in the bottom plate for tube formation assay. (**c**) GSCs were on the upper position, while hBMECs spheroids were placed on the lower plate and cultured in mixed medium (50% containing collagen I and 50% complete EC medium) for angiogenic sprouting assay. (**d**) GSCs were cultured on laminin in the lower plate, and primary GBM cells were located in the upper insert for migration assay. (**e**) GSCs were seeded onto the upper location, and primary GBM cells were cultured on Matrigel in the lower plate for tube formation assay. (**f**) GSCs were cultured on the upper insert, and primary GBM cells were cultivated at bottom.

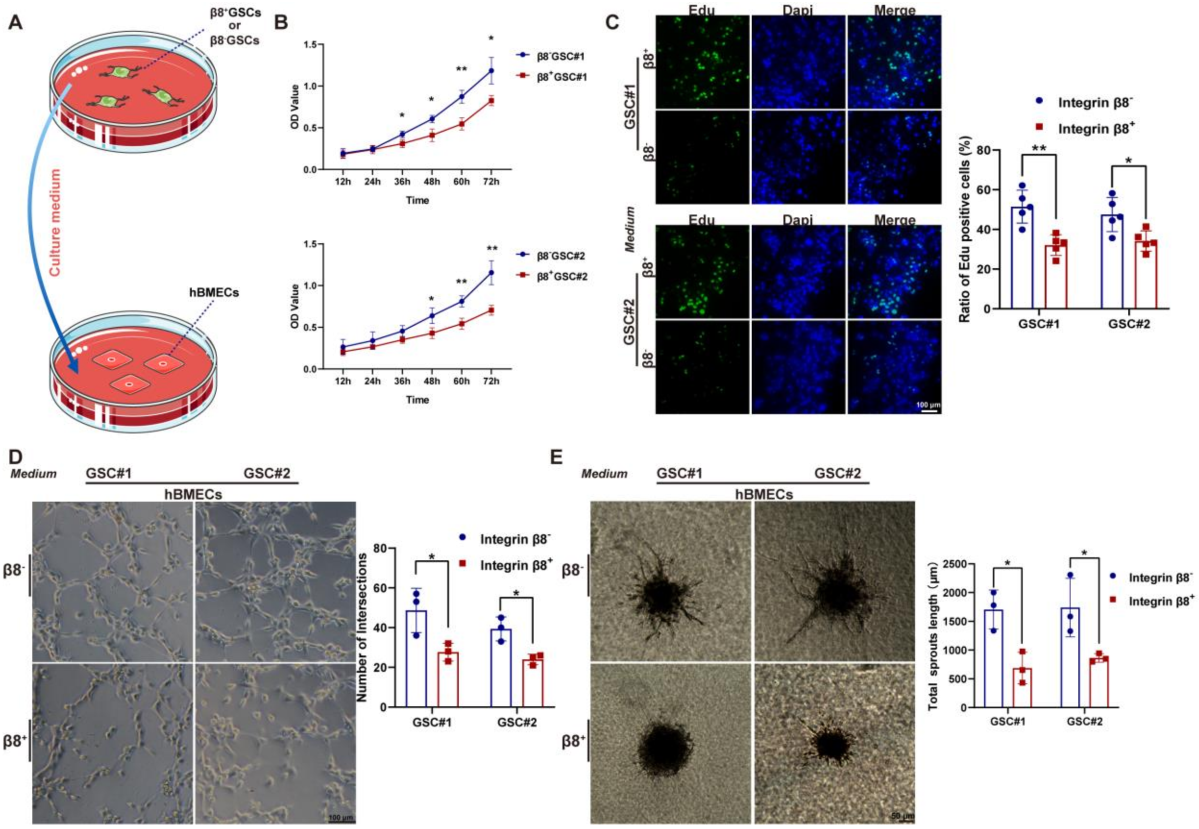


**Figure S4. Conditioned medium of GSCs exhibit anti-angiogenic effect in brain microvascular endothelial cells.** (**a**) GSCs were cultured in serum-free DMEM/F12 medium for 24. Culture medium were collected and for hBMECs cultivation. (**b**) hBMECs were cultivated in conditioned medium derived from $\beta8^+$ or $\beta8^-$ integrin GSC#2 for 24h. And proliferative ability was measured via CCK-8 assay. (**c**) hBMECs were cultured under the same condition and Edu assay was performed. (**d**) hBMECs were cultivated under the same condition, and tube formation assay on Matrigel was assessed. (**e**) hBMECs were cultivated under the same condition, and spheroid-based sprouting assay was performed.

## Original Blotting

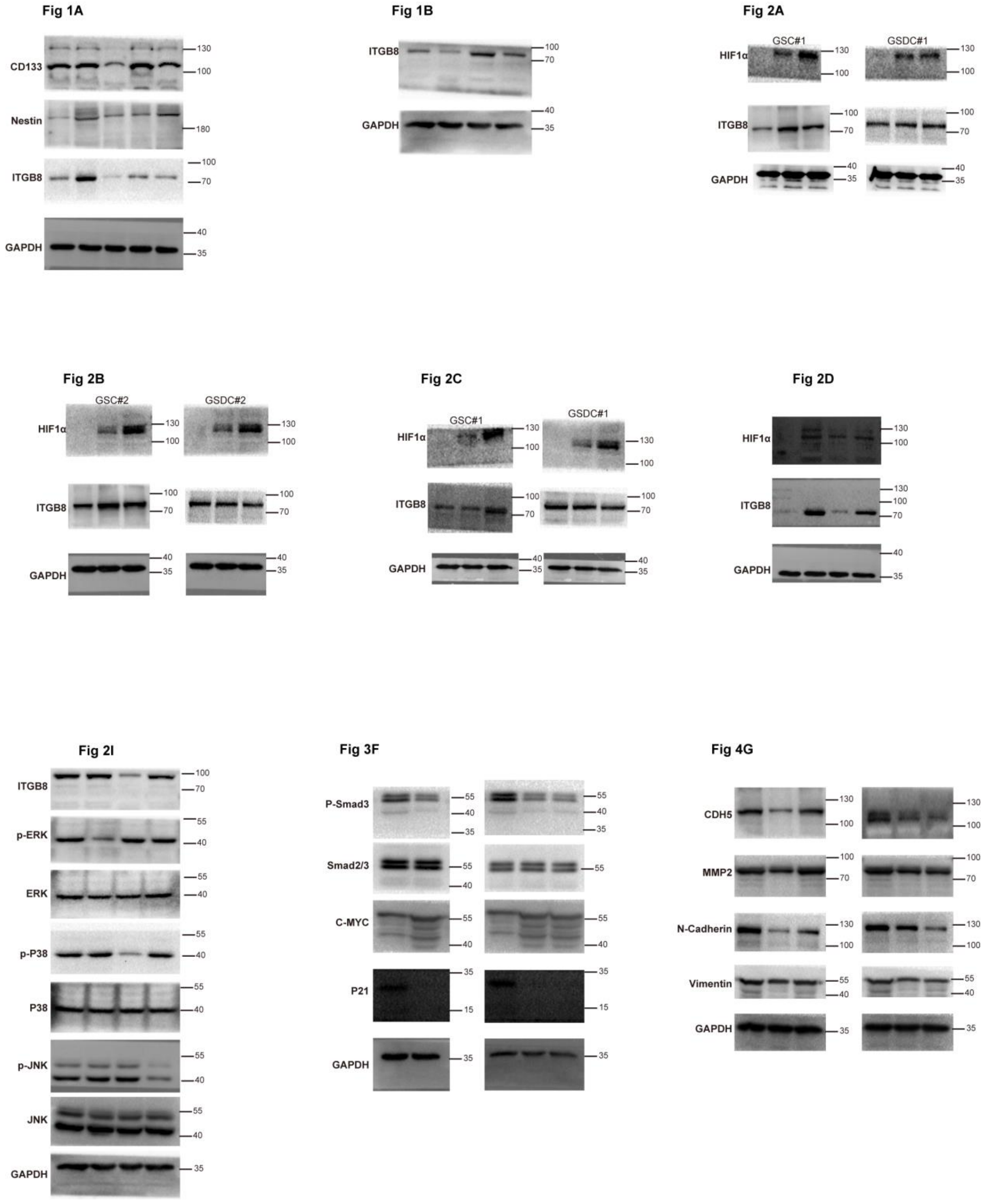


**Figure S5. Uncropped scans of the immunoblots (Fig 1, 2, 3, 4).**

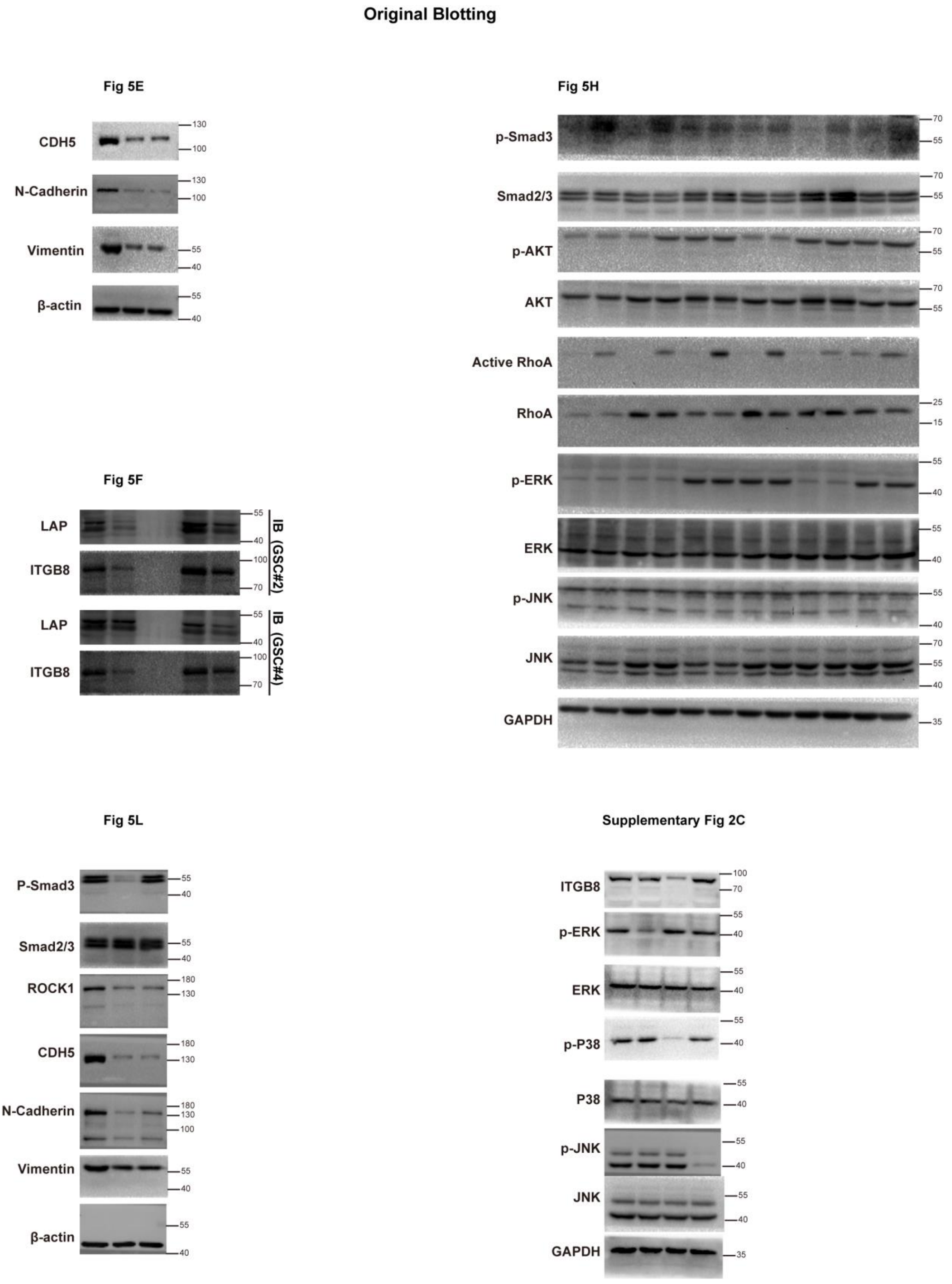


**Figure S6. Uncropped scans of the immunoblots (Fig 5, Supplementary Fig 2).**

Supplementary Table 1

| Clinicopathological Data | ITGB8 expression, n | | | | | |
|---|---|---|---|---|---|---|
| | n | (-) | (+) | (++) | (+++) | P |
| **Gender** | | | | | | 0.368 |
| Male | 51 | 8 | 10 | 24 | 9 | |
| Female | 22 | 1 | 5 | 9 | 7 | |
| **Age(years)** | | | | | | |
| <40 | 22 | 3 | 4 | 12 | 3 | 0.598 |
| ⩾40 to <60 | 46 | 5 | 9 | 19 | 13 | |
| ⩾60 | 5 | 1 | 2 | 2 | 0 | |
| **Tumor Size（cm)** | | | | | | |
| <5 | 31 | 2 | 5 | 19 | 5 | 0.115 |
| ⩾5 | 42 | 7 | 10 | 14 | 11 | |
| **VM** | | | | | | |
| (-) | 27 | 7 | 8 | 9 | 3 | 0.008 |
| (+) | 46 | 2 | 7 | 24 | 13 | |

Supplementary Table 2

| Antibody | Manufacturer | Catalog No. | Usage |
|---|---|---|---|
| β8 Integrin | Genetex | GTX64493 | WB, IHC, IF |
| β8 Integrin | LSBio | LS-E16568 | Blocking |
| CD133 | Genetex | GTX100567 | WB, IHC, IF |
| Nestin | CST | #33475 | WB, IHC, IF |
| HIF-1α | CST | #36169 | WB, IHC, IF |
| Smad2/3 | Genetex | GTX111123 | WB |
| p-Smad3 | Genetex | GTX64209 | WB, IHC, IF |
| C-Myc | CST | #18583 | WB |
| P21 | CST | #2947 | WB |
| CDH5 | Biolegend | 348501 | WB, NE |
| N-Cadherin | CST | #13116 | WB, IHC, IF |
| MMP2 | CST | GTX104577 | WB, IHC |
| Vimentin | Zen Bio | 201158 | WB |
| latent TGFβ | R&D Systems | AF-246-SP | WB, IF |
| ROCK1 | Genetex | GTX113266 | WB, IHC, IF |
| AKT | CST | 9272 | WB |
| p-AKT | CST | 4060 | WB |
| ERK | CST | 4695 | WB |
| p-ERK | CST | 4370 | WB |
| JNK | Proteintech | 10023-1-AP | WB |

| | | | |
|---|---|---|---|
| p-JNK | Affinity | AF3318 | WB |
| P38 | Abcam | ab31828 | WB |
| p-P38 | Abcam | ab47363 | WB, IHC, IF |
| SP1 | GeneTex | GTX110593 | WB |
| GAPDH | Proteintech | HRP-60004 | WB |

| **qRT-PCR Primers** | Forward Sequence (5' to 3') | Reverse Sequence (5' to 3') |
|---|---|---|
| ITGB8 | TCAGTTGATTCAATAGAATACC | CTGTGTATATGAATTTTAGCG |
| GAPDH | GGTGGTCTCCTCTGACTTCAACA | GTTGCTGTAGCCAAATTCGTTGT |

## Supplementary Methods

## Materials and methods

### GBM specimen and cell culture

Brain tumor samples were obtained from consenting patients diagnosed as GBM. Glioblastoma tissues were enzymatically digested and GBM stem cells were cultured in DMEM/F12 (Gibco, USA) medium supplemented with EGF (20 ng/ml, Peprotech, USA), bFGF (20 ng/ml, Peprotech) and B27 (1:50, Gibco). GSCs cell expansion was carried out using both adherent and suspension culture methods. In adherent culture system, GSCs were seeded into flask pre-coated with laminin (10μg/ml, Gibco) and cultivated in serum-free medium. While in suspension culture system, stem cell spheres were generated and propagated as previously described. GSCs used in this study were in passages 2 to 10.

73 paraffin-embedded GBM samples and corresponding clinicopathological data were collected from patients undergoing surgical operation in the department of neurosurgery at Zhujiang hospital from 2013 to 2017.

U87, U251 and A172 cells were purchased from Chinese Academy of Sciences Cell Bank (Shanghai, China). Primary glioma cells G4, G10, G12 were isolated from GBM tissues previously [1]. Primary cells and cell lines were cultured in high glucose DMEM medium (Gibco) supplemented with 10% FBS (Gibco). HBMECs were purchased from ScienCell (Carlsbad, USA) and cultured in supplemented endothelial growth medium (EGM-2, Lonza, Walkersville, MD, USA).

### Reagents, plasmids construction and siRNA

ITGB8 cDNA and plasmids construction, as well as siRNAs for target genes were designed and provided by Sangon Biological Engineering Technology and Service Co., Ltd.

(Shanghai, P.R. China). PCR primer sequences for IGFB8 and GAPDH were provided in supplementary table S2.

**Real-time PCR (qPCR)**

Total mRNA was isolated from GBM stem cells, and cDNA was synthesized from total mRNA. Gene expression of ITGB8 was measured using SYBR Green qPCR kit according to manufacturer's instruction.

**Sphere formation assay**

Dissociated single GBM stem cells were seeded into 24-well plates and incubated in serum-free medium at 37 ℃ for 7 days. Diameters of 5 randomly selected tumor spheres were measured.

**Cell proliferation assay**

Cell proliferation was measured via using CCK-8 and Edu assay. For CCK-8 assay, tumor cells or hBMECs were seeded in 96-well plates (Costar, Cambridge, MA, USA) at a density of $2 \times 10^3$ cells/well and incubated for 7 days. Viable cells were analyzed with Cell Counting Kit-8 (Dojindo, Kumamoto, Japan) according to the manufacturer's guidelines by using a microplate reader (BioTek, Winooski, VT, USA) at 450 nm. Edu assay was conducted based on manufacturer's protocol, cells were incubated for 4 hours following adding Edu solution and then stained by Edu staining proliferation kit (Abcam, USA).

**Cell migration assay**

Migration assay was performed by using cell culture insert with 8-um pores in 24-well plates (Costar, USA). The lower chamber was filled with 0.5 mL medium containing 10% FBS.

Cells ($1x10^5$) were resuspended in 100 µl serum-free medium and seeded onto the upper chamber. This was followed by incubation at 37 ℃ for 24 h. Migrated cells attached to the bottom surface of the insert were stained with crystal violet. Penetrated cells were quantified by ImageJ.

**Tube formation assay**

Tube formation assay of glioma cells and hBMECs was carried out as previously described [1]. Growth factor reduced Matrigel (BD Biosciences, USA) was added to the wells of 96-well plate evenly and incubated at 37 ℃ for 30 min. Cells were then digested and suspended and seeded onto the surface of Matrigel at $2 \times 10^4$ cells/well. After incubation at 37 ℃ for 24h, tube formation was photographed. Tubules were quantified and analyzed by ImageJ software. Intersection quantification of tubules was counted using the Angiogenesis Analyzer-function in the ImageJ toolbar. Intersection of the network was defined as junctions with 3 neighbors.

**Endothelium spheroid-based sprouting angiogenesis assay**

In vitro angiogenesis assay was performed according to methods previously published by Korff and colleagues with minor modification [2]. Briefly, dissociated hBMECs were suspended in EGM-2 medium containing 0.25% methylcellulose and seeded on Low attachment 96-well plates (Corning Inc., USA). Endothelial cells (1000 cells per well) tightly bound to generate a single spheroid after 48h. These spheroids were spun down and re-suspended with 2ml EGM-2 medium supplemented with 20% FBS, followed by the addition of equal amount of Collagen I solution (Enzo life Sciences, USA) and eventually transferred to 24-well plates. The spheroids were incubated for 24h at 37 ℃ and imaged using a phase-contrast microscope. Angiogenesis ability was evaluated by counting the sprout number using NeuronJ plugin of ImageJ software.

**Immunological analysis**

Human TGF-beta1 ELISA kit (Proteintech) was used to measure the concentration of TGF-beta1 from GSCs-derived culture media according to manufacturer's instruction. GSCs were cultured in Low attachment 6-well plates with 2 mL serum-free medium for 72 h, supernatants were then collected for measurement. Experiments were performed in triplicate.

**Immunoblot Analysis and Immunoprecipitation and RhoA-GTP assays**

The immunoblot and immunoprecipitation assay were performed as described before [1]. lysates from certain cells were subjected to immunoblot analysis using antibodies which is listed in supplementary table.

**Tissue Immunohistochemical and Immunofluorescence staining**

Tissue IHC and IF staining were performed as previously reported. Briefly, specimens of surgical GBM tissues and xenograft samples were fixed, embedded and sectioned followed by immuno-staining. Antibodies used for targeted proteins were listed in supplementary table. CD34-PAS histochemical double staining for VM detection was performed according to methods previously published [3].

The results of ITGB8, CDH5, N-Cadherin and MMP2 IHC analysis were evaluated based on Sun's protocol [4]. The staining intensity was scored as follows: 0 for no staining, 1 for weak intensity, 2 for moderate intensity, 3 for strong intensity. While the percentage of immunostaining was rated on a scale from 0 to 3 for no positive area, < 25% of positive area, 25% to 50% positive area and > 50% positive area respectively. The composite IHC score (0, 1, 2, 3, 4, 6, 9) was calculated via multiplying the staining intensity score by the percentage score. Additionally, ITGB8 expression was grouped as negative (-, score = 0), weakly

positive (+, score = 1 or 2), moderately positive (++, score = 3 or 4) and intensely positive (+++, score = 6 or 9).

**Luciferase reporter assay**

Luciferase reporter assay was carried out according to the protocol described previously [5]. Luciferase activity was measured using dual-luciferase reporter assay kit with Renilla luciferase activity as control (Promega, Mannheim, Germany).

***In vivo* xenograft assay**

Five to eight-week-old Balb/c male mice were purchased from the Central Animal Facility of Southern Medical University. Fractionated β8+ or β8- GSCs cells (1 ×105 cells in 0.1 ml PBS) stably transfected with mCherry-LUC vector were orthotopically injected into the brain of Balb/c nude mice according to Ozawa's instruction [6]. The growth of brain tumors was monitored through an *in-vivo* imaging system (IVIS Lumina II, Caliper, USA). The tumor-bearing mice were sacrificed 30 days after implantation and subjected to immunohistochemical or immunofluorescent analysis. The protocol has been registered and approved by the Animal Care and Use Committee of Southern Medical University.

**Vasculature Quantification**

Vasculature quantification was measured according to the method previously reported [7]. Three random specimens from each xenograft sample were subjected to CD34, mCherry and lectin (i.v.) staining. $Lectin^+/mCherry^+$ lumens stand for VM vessels and $CD34^+$ lumens stand for regular endothelium-based vessels. ImageJ software was used for vessel density quantification.

**Statistical analysis**

All statistical analyses in this study were performed using Prism 6.0 (GraphPad Software Inc., USA) and R software. Data were expressed as Mean ± SD. Sample size for each study was determined based on literature documentation of similar well-characterized experiments. Statistical significance was assessed by Student's t test or one-way ANOVA with Bonferroni correction for multiple comparisons. P value smaller than 0.05 was considered statistically significant.


1 Liu Y, Li F, Yang YT, Xu XD, Chen JS, Chen TL *et al.* IGFBP2 promotes vasculogenic mimicry formation via regulating CD144 and MMP2 expression in glioma. *Oncogene* 2019; **38**: 1815–1831.

2 Korff T, Augustin HG. Tensional forces in fibrillar extracellular matrices control directional capillary sprouting. *J Cell Sci* 1999; **112 ( Pt 1**: 3249–3258.

3 Wei X, Chen Y, Jiang X, Peng M, Liu Y, Mo Y *et al.* Mechanisms of vasculogenic mimicry in hypoxic tumor microenvironments. *Mol Cancer* 2021; **20**: 7.

4 Sun Y, Yokoi K, Li H, Gao J, Hu L, Liu B *et al.* NGAL expression is elevated in both colorectal adenoma-carcinoma sequence and cancer progression and enhances tumorigenesis in xenograft mouse models. *Clin Cancer Res* 2011; **17**: 4331–4340.

5 Wei C, Yang C, Wang S, Shi D, Zhang C, Lin X *et al.* Crosstalk between cancer cells and tumor associated macrophages is required for mesenchymal circulating tumor cell-mediated colorectal cancer metastasis. *Mol Cancer* 2019; **18**: 64.

6 Ozawa T, James CD. Establishing intracranial brain tumor xenografts with subsequent analysis of tumor growth and response to therapy using bioluminescence imaging. *J Vis Exp* 2010. doi:10.3791/1986.

7 Zhang L, He L, Lugano R, Roodakker K, Bergqvist M, Smits A *et al.* IDH mutation status is associated with distinct vascular gene expression signatures in lower-grade gliomas. *Neuro Oncol* 2018; **20**: 1505–1516.
